# Vessel Formation Is Induced Prior to the Appearance of Cartilage in BMP-2-Mediated Heterotopic Ossification

**DOI:** 10.1359/jbmr.091031

**Published:** 2009-10-17

**Authors:** Christine Fouletier Dilling, Aya M Wada, Zawaunyka W Lazard, Elizabeth A Salisbury, Francis H Gannon, Tegy J Vadakkan, Liang Gao, Karen Hirschi, Mary E Dickinson, Alan R Davis, Elizabeth A Olmsted-Davis

**Affiliations:** 1 Center for Cell and Gene Therapy, Baylor College of Medicine Houston, TX, USA; 2 Departments of Molecular Physiology and Biophysics and Medicine, Baylor College of Medicine Houston, TX, USA; 3 Department of Pathology, Baylor College of Medicine Houston, TX, USA; 4 Department of Pediatrics, Pediatrics-Nutrition, Baylor College of Medicine Houston, TX, USA; 5 Department of Pediatrics, Hematology-Oncology, Baylor College of Medicine Houston, TX, USA

**Keywords:** bone morphogentic protein type 2, heterotopic ossification, vessel formation

## Abstract

Heterotopic ossification (HO), or endochondral bone formation at nonskeletal sites, often results from traumatic injury and can lead to devastating consequences. Alternatively, the ability to harness this phenomenon would greatly enhance current orthopedic tools for treating segmental bone defects. Thus, understanding the earliest events in this process potentially would allow us to design more targeted therapies to either block or enhance this process. Using a murine model of HO induced by delivery of adenovirus-transduced cells expressing bone morphogenetic protein 2 (BMP-2), we show here that one of the earliest stages in this process is the establishment of new vessels prior to the appearance of cartilage. As early as 48 hours after induction of HO, we observed the appearance of brown adipocytes expressing vascular endothelial growth factors (VEGFs) simultaneous with endothelial progenitor replication. This was determined by using a murine model that possesses the VEGF receptor 2 (Flk1) promoter containing an endothelial cell enhancer driving the expression of nuclear-localized yellow fluorescent protein (YFP). Expression of this marker has been shown previously to correlate with the establishment of new vasculature, and the nuclear localization of YFP expression allowed us to quantify changes in endothelial cell numbers. We found a significant increase in Flk1-H2B::YFP cells in BMP-2-treated animals compared with controls. The increase in endothelial progenitors occurred 3 days prior to the appearance of early cartilage. The data collectively suggest that vascular remodeling and growth may be essential to modify the microenvironment and enable engraftment of the necessary progenitors to form endochondral bone. © 2010 American Society for Bone and Mineral Research.

## Introduction

Endochondral bone formation is thought to proceed through an ordered series of events starting with the proliferation and “condensation” of presumptive mesenchymal cells to form avascular cartilage. Hence it is presumed that the lack of vasculature and associated cellular replication creates the hypoxic environment necessary for chondrogenic differentiation. Using a murine model that possesses the VEGF receptor 2(Flk1) promoter containing an endothelial cell enhancer driving the expression of YFP,(1) we confirmed recent data from our laboratory using a model of heterotopic ossification that suggested that vessels may play an essential role in the induction of chondrogenesis.(2)

It has been well established that vessel formation plays a key role in late events during the process of bone formation. Vessels invade the perichondrium and hypertrophic zone and are required for the replacement of cartilage by bone.(3) The angiogenic factor vascular endothelial growth factor (VEGF) promotes vascular invasion via specific receptors, including Flk1 (VEGF receptor 2) expressed in endothelial cells, in the perichondrium or surrounding tissue.(4,5) These events of cartilage matrix remodeling and vascular invasion are necessary for the migration and differentiation of osteoblasts and osteoclasts, which remove mineralized cartilage matrix and replace it with bone. However, much less is known about the role of vessel formation prior to the appearance of the precartilage tissue.

During normal wound repair, a series of cell signaling events is induced by the hypoxic state of the tissues, resulting in upregulation of hypoxia inducible factor (HIF1), which, in turn, upregulates a series of factors including several VEGFs (A, B, and D), leading to vessel formation. Hypoxia-induced angiogenesis has been proposed to be necessary for creating specialized vessels that facilitate progenitor homing and engraftment into damaged tissues.(6) Little is known about whether such a process plays a key role in the repair of bone.

Using a model of de novo bone formation to identify the earliest events in this process, we have demonstrated that myelomesenchymal stem cells are recruited to the tissues to form the early cartilage.(7) One of the earliest events in this model is the appearance of brown adipocytes. These cells are capable of using their uncoupled aerobic respiration to reduce localized oxygen tension and effectively pattern the newly forming cartilage condensations.(8) This is consistent with in vitro data showing that bone marrow–derived mesenchymal stem cells can undergo chondrogenesis in the presence of bone morphogenetic protein 2 (BMP-2) and low oxygen.(9) We also observed the appearance of vessels lining the edges of the perichondrial region, separated only by brown adipose tissue, suggesting that perhaps the reduction in oxygen tension coordinately activates new vessel formation in the region.(8) Thus these progenitors may indeed be recruited to the site of new bone formation through the vasculature. In this study we focused on defining this tentative early vessel formation.

To determine this, we chose to employ a transgenic mouse model that expresses the fusion protein human histone H2B with enhanced yellow fluorescent protein (EYFP) (H2B:YFP) in endothelial cells under the regulation of a Flk1 promoter/enhancer fragment (Flk1-H2B::YFP).(1) Recent improvements in genetically encoded fluorescent protein expression in animal models, along with advances in optical imaging and image analysis software, have enabled the analysis of many aspects of tissue development at a cellular level.(10) Previous studies using this transgenic animal indicates that Flk1-H2B::YFP expression is restricted to endothelial cells of smaller and/or newly forming vessels,(8) thus providing a mechanism for quantification of new vessels.

Here we demonstrate new vessel formation within the tissues prior to the appearance of the presumptive cartilage. Quantification of the number of endothelial cells shows that one of the first steps of bone formation is to induce additional endothelial cell proliferation. Histologic analysis shows that increases in endothelial cell numbers are evident just prior to the influx of chondrocytic progenitors. Immunohistochemical analysis of the tissues prior to the mesenchymal condensations revealed a rapid and transient expression of VEGF-A and -D from the brown adipocytes. The data collectively suggest that the brown adipocytes may play a key role in establishing patterning of the cartilage via regulation of oxygen tension within the tissues through induction of both aerobic respiration and early angiogenesis.

## Materials and Methods

### Cell culture

A murine C57BL/6-derived cell line (MC3T3-E1) was obtained from American Type Culture Collection (Manassas, VA), propagated in α modified essential medium (α-MEM) supplemented with 10% FBS (Hyclone, Logan, UT, USA), 100 U/mL penicillin, 100 µg/mL streptomycin, and 0.25 µg/mL amphotericin B (Life Technologies, Inc., Gaithersburg, MD, USA). Briefly, the cells were grown in DMEM supplemented as described earlier and cultured at a subconfluent density to maintain the phenotype. All cell types were grown at 37°C and 5% CO_2_ in humidified air.

Transduction of cells with adenovirus in the presence of GeneJammer adenoviruses

Replication defective first-generation human type 5 adenovirus (Ad5) deleted in regions E1 and E3 was constructed to contain the cDNA for BMP-2 in the E1 region of the viral genome.(11) The virus particle (vp) to plaque-forming unit (pfu) ratios were 55 and 200 for Ad5-BMP-2 and Ad5-empty, respectively, and all viruses were shown to be negative for replication-competent adenovirus.

The C57BL/6 cell line, or MC3T3-E1 (1 × 10^6^), was transduced with Ad5-BMP-2 or Ad5-empty cassette control virus at a concentration of 5000 vp/cell with 1.2% GeneJammer, as described previously.(12)

### Heterotopic bone assay

The transduced cells were resuspended at a concentration of 5 × 10^6^ cells/100 µL of PBS and then delivered through intramuscular injection into the hind limb quadriceps muscle of *Flk1* mice. Animals were euthanized at daily intervals, and hind limbs were harvested, embedded, and stored at −80°C. All animal studies were performed in accordance with standards of the Baylor College of Medicine, Department of Comparative Medicine, after review and approval of the protocol by the Institutional Animal Care and Use Committee (IACUC).

### Histologic analysis and staining analysis

Soft tissues encompassing the site of new bone formation were isolated from the rear hind limbs of the mice. Both the skin and skeletal bone were removed from the tissues prior to freezing. Serial sections (15 µm) were prepared that encompassed the entire tissue (approximately 50 sections per tissue specimen). We then performed hematoxylin and eosin staining on every fifth slide, which allowed us to locate the region containing either our delivery cells or the newly forming endochondral bone. Serial unstained slides were used for immunohistochemical staining (either single- or double-antibody labeling). For double-antibody labeling, samples were treated with both primary antibodies simultaneously, followed by washing and incubation with respective secondary antibodies, used at 1:500 dilution, to which Alexa Fluor 488, 594, or 647 was conjugated. Primary antibodies were used as follows: SMA mouse monoclonal used at 1:200 dilution (Sigma Chemical Company, St Louis, MO, USA), CD31 rat monoclonal used at 1:75 dilution (BD Pharmingen, San Diego, CA, USA), Flk1 goat polyclonal used at 1:100 dilution (R&D Systems, Minneapolis, MN, USA), Ki67 rat monoclonal used at 1:100 (Dako, Carpinteria, CA, UDA), and VEGF-D goat polyclonal used at 1:100 dilution (Santa Cruz Biotechnology, Inc., Santa Cruz, CA, USA). Stained tissue sections were examined by confocal microscopy (LSM 510 META, Zeiss, Inc., Thornwood, NY, USA) using a 20×/0.75NA objective lens.

### Flk1-positive cell quantification in BMP-induced tissues

To quantify the increase in YFP-positive cells in the BMP-induced tissues, frozen sections across these tissues were counterstained with 4,6-diamidino-2-phenylindole (DAPI), and the YFP expression was compared with that obtained in the control tissues. First, a series of low-magnification (5.4× and 12×) bright-field images of a tissue section was taken and overlapped to reconstruct the tissue section using Adobe Photoshop CS3 (San Jose, CA, USA). The reconstructed montage image was used to measure the area of the tissue section using a manual contour-tracing method (Zeiss Axiovision). The area of each of the frozen sections was calculated in a similar manner. Area measurements are used to determine the density of labeled cells, as indicated below.

High-resolution (10×/NA0.45, 1024 × 1024 pixels) dual-channel images of tissue sections nuclear stained with DAPI were taken using a confocal microscope (Zeiss LSM 510 META). In each image, the number of nuclei in the DAPI and YFP channels was counted using a modified watershed segmentation algorithm (FARSIGHT, Farsight Image Segmentation Software, courtsey of Badri Roysam, RPI, Troy, NY), which makes use of both intensity and volume thresholds to distinguish two nuclei as separate. All the nuclei counted using the software were DAPI^+^. The fraction of DAPI-stained nuclei marked by YFP was counted as YFP^+^. The density of YFP^+^ cells in a tissue section was defined as the ratio of the number of YFP^+^ nuclei in the tissue section measured from the high-magnification images to the area of the tissue section measured from the low-magnification images. The density of the YFP^+^ nuclei was calculated for a number of control and BMP-treated tissues at 2 and 4 days after injection. The ratios then were averaged over the various control and BMP-2-treated tissues. The *p* values were calculated using a Student's *t* test.

### Flk1-YFP^+^ cell association analysis

To characterize the cell type(s) that express YFP in the adult muscle tissue, we performed immunoflourescence studies using endothelial cell marker CD31 and Flk1 antibodies; for vascular smooth muscle cells, we used smooth α actin (SMA). Association of cells expressing YFP with immunolabeled cells was analyzed using (FARSIGHT, RPI) and a custom program written in MATLAB (MathWorks, Natick, MA, USA). After identification of each nucleus by DAPI staining, YFP^+^ and YFP^−^ cells then were analyzed for association with the fluorescent signals of each antibody. An intensity threshold was applied to the red channel in each image to identify a cell positive or negative for the immunofluorescent signal. Each identified nucleus and overlapping red channel were counted as CD31^+^, Flk1^+^, or SMA^+^ and then as either YFP^−^ and YFP^+^. Colocalization percentages are shown in the supplemental data section (Table S-1) and described in detail the YFP^+^ cell types. The number of YFP^+^/Ki67^+^ nuclei in an area of the tissue was calculated by adding the YFP^+^/Ki67^+^ in each of the confocal images taken within the area. The area fraction of YFP^+^/Ki67^+^ was defined as the total number of YFP^+^/Ki67^+^ in the images taken within the area divided by the number of images. The area fraction was measured for five different areas, and the average area fraction was calculated for control and BMP-treated tissues for every fifth slide sectioned throughout the entire hind limb. The area fractions of YFP^+^/Ki67^+^ nuclei in the control and the BMP-treated tissues on day 2 were 3.97 ± 2.96 and 6.11 ± 1.76, respectively. The area fractions for day 4 were 5.04 ± 0.72 and 6.41 ± 1.41 in the control and the BMP-treated tissues. Based on the Student's *t* test, the *p* value for the day 2 data was .21, and that for the day 4 data was .10. Taken together, the data support the trend that the YFP^+^/Ki67^+^ population increases on day 2 and day 4 after the BMP treatment.

### qRT-PCR

Nonskeletal tissues (*n* = 4 per group) surrounding the site of injection of the Ad5-BMP-2 or Ad5-control transduced cells were isolated at daily intervals for 7 days and prepared as total RNA using a Trizol reagent (Life Technologies, Carlsbad, CA, USA) in accordance with the manufacturer's specifications. The two groups of RNAs were subjected to qRT-PCR analysis in parallel, and the *C*_*t*_ values obtained normalized to both internal 18S ribosomal RNA used in multiplexing and to each other to remove changes in gene expression common to both the BMP-2 and control tissues by using the method of ΔΔ*C*_*t*_ along with Taqman primers and probes (Applied Biosystems, Carlsbad, CA, USA) as described previously.(8)

## Results

### Upregulation of vessel markers prior to the onset of chondrogenesis

**Fig. 1:**
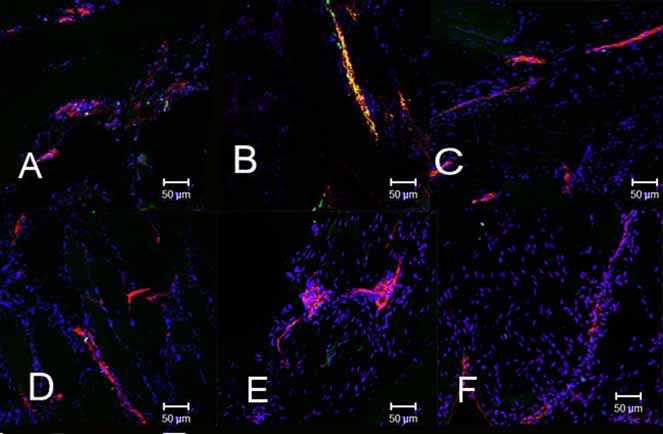
Immunohistochemical analysis of endothelial cell replication in tissues isolated at daily intervals after induction of bone formation with cells expressing BMP-2. (*A–E*) On days 1, 2, 3, 4, and 5, respectively, after injection of BMP-2-producing cells, paraffin sections were prepared and stained with an antibody against Ki67, followed by a secondary antibody conjugated to Alexa fluor 488 (green) mixed with an anti–von Willibrand Factor (vWF) antibody, followed by a secondary antibody conjugated to Alexa fluor 547 (red). (*F*) A representative image, similar staining, taken from tissues isolated from mice injected with cells transduced with a control vector (Ad5-empty).

We have previously described a model of rapid endochondral bone formation(13) in which mineralized bone is observed 7 days after the initial induction with BMP-2. Observation of vessels lining the newly forming perichondrium suggests that vessels may undergo replication prior to chondrogenesis. To confirm this hypothesis, we examined tissues, at 24-hour intervals over the period leading up to chondrogenesis (day 5), for the presence or absence of endothelial cell replication. Figure 1 shows the coexpression of the endothelial cell–specific factor von Willibrand factor (vWF) (red) and Ki67 (green), a marker of cellular replication,(14) in the vessels from tissues that received Ad5-BMP-2-transduced cells starting at 24 hours and going to 5 days (panels *A–E*, respectively). As can be seen in Fig. 1*B*, we did observe overlap of these two markers (yellow) in tissues receiving the Ad5-BMP-2-transduced cells, whereas no replicating endothelial cells were observed in the control tissues (Fig. 1*F*). We did not attempt to quantify the amount and apparent timing of replication using this method because vWF is an extracellular matrix protein. Instead, we employed the Flk1-H2B::YFP model for quantifying endothelial progenitor replication over the course of early bone formation.

### Flk1-H2B::YFP in vessels

We next determined if there was a significant increase in the number of Flk1^+^ endothelial progenitors during bone induction, consistent with new vessel formation, prior to chondrogenesis. We chose to use the Flk1-H2B::YFP mouse model,(1) in which new vessel formation could be readily quantified within the muscle tissues. Flk1 is a VEGF receptor transiently expressed on endothelial cells and is thought to contribute to VEGF-induced endothelial cell replication.(15) Therefore, quantification of the nuclear YFP expression within tissues from animals receiving either Ad5-BMP-2- or Ad5-empty-transduced cells allowed us to to quantify increases in the number of endothelial progenitors within the muscle prior to cartilage formation. We previously quantified the association of Flk1-H2B::YFP mouse model with other endothelial cells markers such as CD31 and found them to be 95% overlapping (see Supplemental Data below). Frozen sections were prepared by serial sectioning from Flk1-H2B::YFP adult hind limb soft tissue (*n* = 4 per group) consisting of three groups, those receiving (1) cells transduced with Ad5-BMP-2, (2) cells transduced with Ad5-empty cassette control virus, and (3) normal mouse muscle. To ensure uniform quantification and adequate sampling, the entire region of soft tissues in the hind limb was sectioned, and approximately every fifth section was analyzed for YFP expression.

**Fig. 2:**
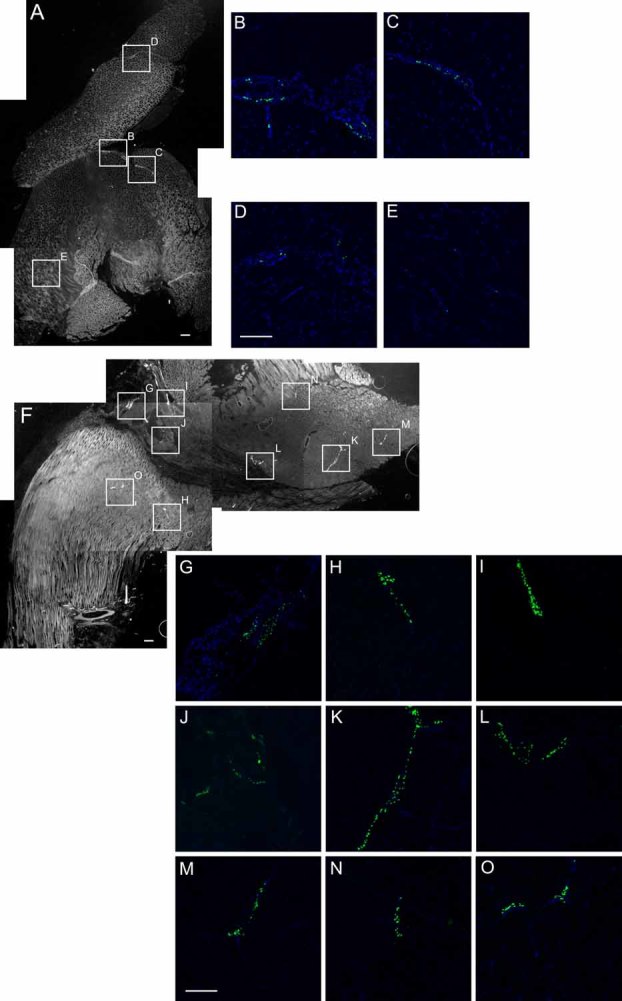
Wide-field and confocal images of whole tissue sections and quantification of Flk1-H2B::YFP cells. (*A*, *F*) Representative montages of low-magnification gray-scale images (1 pixel = 0.003 mm) used for calculating total area for tissue sections. A single representative tissue section is depicted after the entire hind limb muscles that encompassed the injection site were isolated 2 days after receiving an intramuscular injection of cells transduced with either Ad5-empty control vector (*A*) or Ad5-BMP-2 (*B*) and sectioned at 15 µm thickness. Although every fifth section across the entire tissue was analyzed, we show only a single representative image of each type. The corresponding regions with positive YFP signal, shown by the boxed areas, were imaged by confocal microscopy (*B–E*, *G–O*) for counting the YFP^+^ cell numbers.

**Fig. 3:**
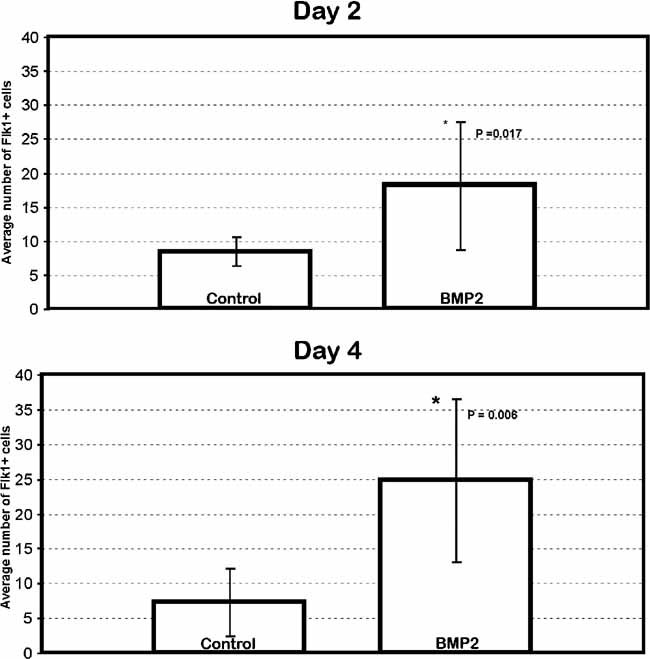
Increase in Flk-H2B::YFP^+^ cells in BMP-2-induced tissue on days 2 and 4. Quantification of Flk1-H2B::YFP^+^ cells within the tissues 2 and 4 days after induction with Ad5-BMP-2-transduced or control cells. YFP^+^ nuclei were counted and reported as a ratio of the total area of the tissue section determined using the wide-field montage. Flk-H2B::YFP^+^ cells were significantly elevated in the tissues receiving BMP-2 compared with controls. The graph depicts the average number of Flk-H2B::YFP^+^ cells in five sections for day 2 control, 7 sections for day 2 BMP, 8 sections for day 4 control, and 6 sections for day 4 BMP. The number of images taken in each section ranged from 4 to 22. *Denotes a significant difference as determined by the Student's *t* test.

To quantify differences in the number of endothelial progenitors, the number of YFP^+^ cells per the total number of DAPI^+^ cells was determined using automated segmentation methods (see Materials and Methods). The total YFP^+^ cells also was quantified per total area of the tissue section to ensure that there was no bias in the fields of view chosen for image analysis (see Materials and Methods). The total area of each tissue section was determined using a montage of images that were collected using wide-field microscopy (Fig. 2*A, F*). As can be seen in Fig. 2, we found Flk1-H2B::YFP^+^ cells in both tissues receiving Ad5-empty-transduced cells (Fig. 2*B–E*) and Ad5-BMP-2-transduced cells (Fig. 2*G–O*). These panels are higher-magnification confocal images of the region within the corresponding white box on the lower-magnification high-resolution wide-field montage of the entire tissues (Fig. 2*A*, control; Fig. 2*F*, BMP-2). The results of the quantification (Fig. 3) shows the average number of Flk1-H2B::YFP^+^ cells on days 2 and 4. Analysis of the entire soft tissue within several mice showed a significant elevation in tissues receiving the Ad5-BMP-2-transduced cells (*p* = .017, day 2, Fig. 3*A* and *p* = .006, day 4, Fig. 3*B*) compared with control on both days 2 and 4. The peak was approximately 2 days after induction of bone formation, with no statistically significant difference between these results and those obtained in tissue sections isolated 4 days after induction.

### Endothelial progenitors undergo replication in tissues receiving Ad5-BMP-2-transduced cells

**Fig. 4:**
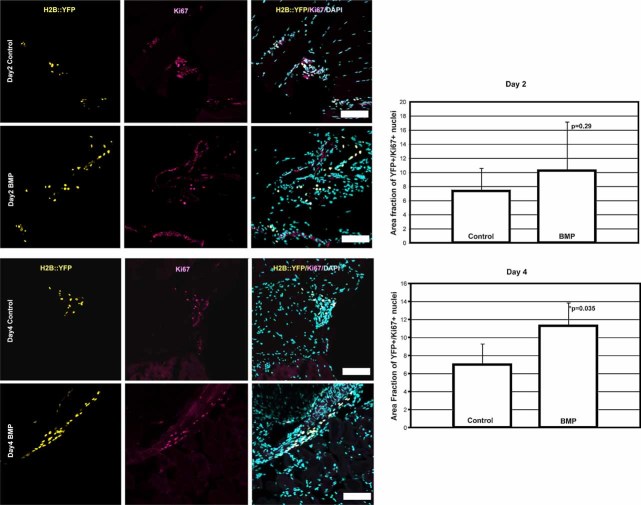
Quantification of YFP^+^ cell proliferation. Representative images of Flk1-H2B::YFP and the cell proliferation marker Ki67. Colocalization of Flk1-H2B::YFP (yellow) and Ki67 (red) was detected in BMP-2-treated and control tissues. Graphs show the total number of YFP^+^/Ki67^+^ cells in the images taken within the area divided by the number of images analyzed. The area fraction was measured for nine at day 2 and five at day 4 BMP and eight at day 2 and four at day 4 control different areas, and the average area fraction was calculated for control and BMP-treated tissues. The area fractions of YFP^+^/Ki67^+^ nuclei in the control and the BMP-treated tissues on day 2 were 7.32 ± 3.26 and 10.20 ± 6.95, respectively. The area fractions for day 4 were 6.97 ± 2.32 and 11.26 ± 2.58 in the control and the BMP-treated tissues. Based on the Student's *t* test, the *p* value for the day 2 data was .29 and that for the day 4 data was .035. Taken together, the data showed significant YFP^+^/Ki67^+^ population increases by day 4 after the BMP treatment, but on day 2 there were no significant differences in dividing YFP cell population between control and BMP-treated tissues.

In the tissues receiving cells transduced with a control adenoviral vector we observed randomly scattered YFP^+^ cells along the vessel structures, whereas in the tissues receiving Ad5-BMP-2-transduced cells we saw clustering of the YFP^+^ cells (Fig. 2). This prompted us to question whether the Flk1-H2B::YFP progenitors could be replicating, so we next quantified the number of Flk-H2B::YFP^+^ cells in these tissues. Representative images used for quantification of YFP^+^ cell proliferation activity are shown in Fig. 4. Replicating endothelial progenitors were defined as nuclei positive for both Flk1-H2B::YFP (yellow) and the cell proliferation marker Ki67 (red; Fig. 4). In both control and treated animals, we also observed proliferating cells positive for Ki67 that did not overlap with Flk1-H2B::YFP. Quantification of cells positive for both Flk1-H2B::YFP and Ki67 (Fig. 4) indicates a large number of replicating endothelial progenitors in both control and BMP-2-treated tissues at 2 days after induction. Therefore, the percentage of dual-positive cells was not significant at this early time point compared with control. However, by 4 days after induction with BMP-2, we observed much fewer replicating Flk1-H2B::YFP cells in the control group and significantly more in the experimental group (Fig. 4). This difference was found to be statistically signifcant.

### Vascular endothelial growth factor (VEGF) mRNA expression

**Fig. 5:**
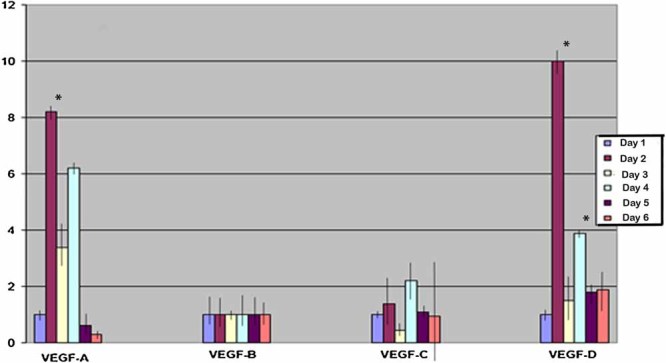
Expression of VEGF-D during the early stages of endochondral bone formation. Results of qRT-PCR analysis of *VEGF-A*, *-B*, *-C*, and *-D* mRNA levels in tissues surrounding the lesional site that received either the Ad5-BMP-2- or Ad5-empty-transduced cells isolated at daily intervals for up to 7 days after initial injection. Four biologic replicates were run in triplicate, and the averages were normalized against an internal standard (ribosomal RNA). The samples receiving Ad5-BMP-2-transduced cells then were compared with those obtained from the tissues receiving cells transduced with Ad5-empty cassette virus. Therefore, the graph depicts the fold changes in VEGF RNAs in the BMP-2 samples over time compared with control tissues. Error bars depict ± 1 SD unit. *Denotes samples that had a statistically significant (*p* < .05) difference from all other samples by the ANOVA test.

Endothelial progenitor replication appeared to start within 48 hours of induction with BMP-2. This correlated with a significant elevation in *VEGF-D* [also termed *fos-induced growth factor* (*FIGF*)] and *VEGF-A* RNA expression (Fig. 5). Figure 5 shows the changes in VEGF mRNA expression from day 1 after injection of Ad5-BMP-2-transduced cells until day 6, as determined by real-time RT-PCR (qRT-PCR). Both *VEGF-A* and *VEGF-D* mRNA expression was significantly increased on days 2 and 4 after induction of bone formation. *VEFG-B* and *VEGF-C* RNA, however, remained on the same level throughout the time course. Although the data cannot differentiate between expansion of cells expressing VEGF-A and -D and elevated transcription within cells residing in the area, the results suggest that these potent endothelial growth factors are rapidly and transiently increased within the site of new bone formation prior to the onset of cartilage.

### Role of brown adipose in vessel formation

The data collectively suggest that vessel replication is occurring simultaneously with elevated expression of VEGFs within the tissues. Since one of the earliest events observed in our model is the recruitment and expansion of brown adipocytes,(8) we next chose to determine if these cells might be expressing the VEGFs.

**Fig. 6:**
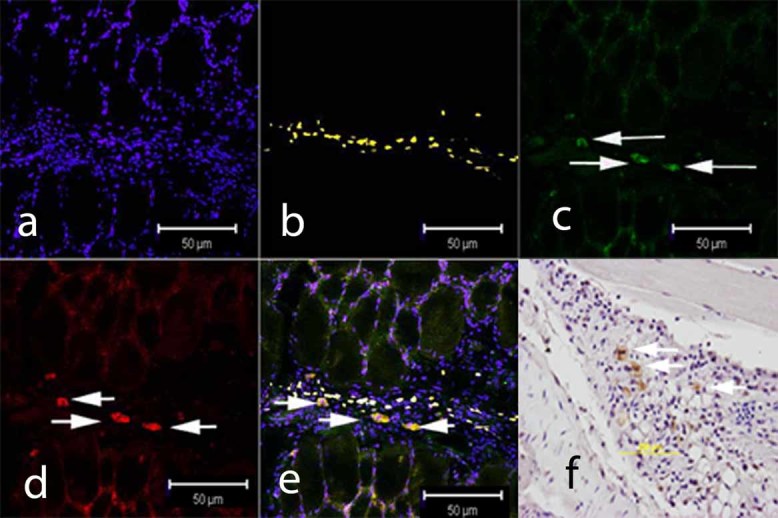
Immunohistochemical staining for brown adipocytes expressing VEGF-D (green, *c*) in tissues isolated from the Flk1-H2B::YFP mice 4 days after receiving MC3T3 cells transduced with Ad5-BMP-2. Brown adipocytes were identified as cells expressing uncoupling protein 1 (UCP 1; red, *d*) and yellow (*b*) represents the Flk-yfp^+^ endothelial cells within the muscle. The tissues also were stained with VEGF-D antibodies (*c*) and counterstained with DAPI (blue, *a*), which stains the nucleus of cells. A merger of these stains (UCP-1, VEGF-D, and YFP) is shown in panel *e*. In panel *f*, a paraffin section taken 4 days after injection of BMP-2-producing cells was stained with an antibody against UCP1, and staining was visualized using 3,3'-diaminobenzidine (DAB) as described previously.(8) No staining was observed on a paraffin section taken 4 days after injection of cells transduced with the empty control vector Ad5-HM4 (data not shown).

Immunohistochemical analysis of Flk1-H2B::YFP tissues that received either Ad5-BMP-2- or Ad5-empty-transduced cells showed colocalization of VEGF-D (green, Fig. 6*c*) and the brown adipocyte-specific marker uncoupling protein 1 (UCP1; red, Fig. 6*d*) (day 2). As can be seen in Fig. 6*e*, expression of UCP1 overlaps expression of VEGF-D in cells that are adjacent to the Flk1-H2B::YFP^+^ endothelial progenitors, suggesting that the brown adipocytes may be contributing to the new vessel formation. We observed additional fluorescence within the surrounding muscle that appears to be punctate and not cell-associated. This staining may represent VEGF-D protein secreted in the tissues. To confirm the cell-specific expression of VEGF-D in the brown adipocytes, we performed additional immunostaining (Fig. 6*f*). Positive expression of VEGF-D (brown staining) was observed only in the brown adipocytes, again suggesting that these cells may play a role in the regulation of new vessels.

## Discussion

Similar physiologic steps lead to bone formation during embryonic development and in adult organisms, for instance, in fracture repair or heterotopic ossification. In both cases, bone formation begins with mesenchymal condensation and ends with maturation of the growth plate, recruitment of osteoblasts, and the production of bone. Vascularization has been shown to play a critical role in this process through infiltration into cartilage to form vascularized bone.(16) Here we present data that show that vessels play a much earlier role in patterning of the cartilage and bone. The results show the presence of new vessel formation prior to the onset of mesenchymal condensation and cartilage.

We have previously reported the presence of brown adipocytes within the tissues 2 days after the initial induction. We also have shown that these cells regulate localized oxygen tension through their unique metabolism.(8) In this study we extend our knowledge of the functional role of brown adipocytes to include their rapid and transient expression of the potent angiogenic factors VEGF-A and -D. Interestingly, a similar rapid and transient expression of VEGF-D also has been demonstrated in limb development and has been shown to be critical for patterning.(17) We observed a biphasic expression pattern for VEGF-A and -D, suggesting multiple roles for this factor in bone formation. The second peak of expression correlates nicely with the transition of cartilage to bone formation, which has been clearly documented.(16,18) However, the first phase is less well studied, and in our model it appears to correlate with the establishment of new vessels just prior to the onset of chondrogenesis. Zelzer and colleagues also reported a similar biphasic expression of VEGF-A during embryonic bone formation.(19) In these studies, the authors showed two functional roles for VEGF-A, one prior to cartilage and one during the transition of cartilage to bone, similar to our own observation in our model. The data collectively suggest that the brown adipocytes may induce the synthesis of new vessels as a component for patterning the newly forming cartilage. In the proposed model, the brown adipocytes induce new vessels, facilitating the recruitment of chondrogenic precursors, while at the same time lowering localized oxygen tension to allow for chondrogenic differentiation. In support of this mechanism, we show in this study the presence of brown adipocytes expressing VEGF-D only in areas adjacent to our newly expanding vessels, as marked by Flk1-H2B::YFP.

Using a model of rapid endochondral bone formation, we show the immediate expansion of vessels within the tissues in response to delivery of BMP-2. Although BMP-2 and -4 play a critical role in the patterning of cartilage and bone in the embryo,(20) much evidence now links the BMPs to a host of other earlier physiologic functions, including vascularization of the early embryo.(21) Thus it may not be surprising that the earliest stage of bone formation in our model is the induction of new vessel formation.

On BMP-2 stimulation, the Flk-1-H2B::YFP endothelial progenitors expand as the total number of positive cells per tissue area increases. The Flk1-H2B::YFP^+^ cells are clustered along individual vessels, suggesting that these vessels are extending or remodeling in response to BMP-2. At this point, we cannot determine whether this increase occurs via replication of tissue-resident endothelial progenitors or the recruitment of progenitors to the site of new bone formation. Our data suggest that the expansion of these progenitors, at least in part, is due to replication because we observed an increase in the area of replicating endothelial cells within the tissues receiving Ad5-BMP-2-transduced cells on day 4 compared with control tissues. However, we cannot rule out the possibility that at least some of these cells are recruited from either the circulation or surrounding tissues. Interestingly, their were significant clusters of replicating Flk1-H2B::YFP cells on day 2 in tissues receiving both the Ad5-BMP-2- and the Ad5-empty-transduced cells, suggesting that perhaps the initial inflammatory reponse may be somewhat masking the significance of the replication at this early time point. Alternatively, the increase in replication of the Flk1-H2B::YFP cell population at 4 days after induction of bone formation may represent the need for vascularization to recruit new chondro-osseous progenitors because this coincides with the appearance of these cells within the tissues.(22) However, recruitment from the surrounding tissue is equally likely because recently Kaplan and colleagues(34) showed local stem and progenitor cell contribution to heterotopic bone formation in a murine model of stem cell transplantation, and this process may require new vessel formation for establishment of these cells.

VEGFs have been shown to be essential to expansion of both endothelial cells and vascular smooth muscle cells that assemble to form the vessel structure. Although VEGF-A most commonly has been shown to be responsible for angiogenesis in most systems, recent studies in murine muscle have found VEGF-D to be an extremely potent angiogenic factor.(23) This family member is better known for its critical role in the expansion of lymphatic vasculature.(23) In our model we see both factors highly expressed in the tissues receiving the Ad5-BMP-2-transduced cells compared with those receiving control cells. Again, the rapid but transient elevation in VEGF expression suggests that these factors may be driving the endothelial cell replication. Knockout studies have confirmed that BMPs regulate vasculogenesis during embryonic development.(24) Functional deletion of BMP-4 and the BMP I receptor in mice leads to impaired mesoderm precursors required for vascular development.(25,26) It also has been shown that addition of BMP-neutralizing antibodies or noggin suppresses endothelial cell formation during development, whereas addition of rhBMP-4 promotes it.(27)

We and others have recently shown the chondrocyte to be of myeloid origin, and it circulates to the site of new bone formation.(22,28) These cells then must recruit and pass from the vessels into the tissues, through a process known as *extravasation*.(29) This process has been shown to require small vasculature that has a reduced blood flow.(29) Thus it is conceivable that brown adipocytes express the VEGFs to form new vessels that are capable of permitting recruitment of chondrocytic progenitors to the correct location for endochondral bone formation. Since vascular invasion of the growth plate has been well documented to precede the recruitment of osteoblast progenitors to form the new bone,(16,18,29,30) it would not be surprising to have an earlier phase of this process that recruited the chondrocytic progenitors. We have shown previously that the brown adipocytes are capable of inducing hypoxia in the local environment, which in the presence of BMP-2 has been shown to induce chondrogenesis.(8) Thus we propose that the brown adipocytes are capable of patterning the newly forming cartilage by inducing new vessel formation while simultaneously removing oxygen through uncoupled aerobic respiration. Once the progenitors differentiate into chondrocytes, they then express a number of anti angiogenic proteins to prevent in the growth of new vessels, thus momentarily attenuating this early wave of angiogenesis.(31–33,35) Thus the results presented in this study extend our knowledge about the critical role vascularization plays not only in bone formation but also in cartilage formation as well. The data collectively show a novel process for patterning of new endochondral bone in adult organisms. Further, this is one of the first studies that attempts to understand the biology of tissue engineering of cartilage. Surprisingly, one of the critical components we have identified is contradictory to our current dogma that cartilage does not require vessels. This study suggests that brown adipose may play a pivotal role in establishing new vessels, essential for recruitment of chondrogenic progenitors and patterning of the tissues. These findings ultimately may play an important role in our efforts to replace damaged cartilage through tissue engineering.
